# Supplementary material for: Multifield-Controlled Terahertz Hybrid Metasurface for Switches and Logic Operations
Source: Nanomaterials (Basel). 2022 Oct 26;12(21):3765. doi: 10.3390/nano12213765 (PMC9658003; doi:10.3390/nano12213765)
Supplement: Supplementary file 1 [file nanomaterials-12-03765-s001.zip › nanomaterials-1859711-supplementary.pdf]

Supplementary Materials

Multifield-Controlled Terahertz Hybrid Metasurface for Switches and Logic Operations

Xilai Zhao [1,†], Yanan Jiao [2,†], Jiangang Liang [1,*], Jing Lou [1], Jing Zhang [1], Jiawen Lv [3], Xiaohui Du [2], Lian Shen [4], Bin Zheng [4] and Tong Cai [1,4,*]

1 Air and Missile Defense College, Air Force Engineering University, Xi'an 710051, China
2 Department of General Surgery, First Medical Center, Chinese People's Liberation Army (PLA) General Hospital, Beijing 100024, China
3 China Nuclear Engineering Consulting Corporation, Beijing 100024, China
4 Interdisciplinary Center for Quantum Information, State Key Laboratory of Modern Optical Instrumentation, Zhejiang University Hangzhou Global Scientific and Technological Innovation Center, Zhejiang University, Hangzhou 310027, China
* Correspondence: cat-liang1975@163.com (J.L.); caitong326@zju.edu.cn (T.C.)
† These authors contributed equally to this work.

S1: Discussion of available fabrication process of the proposed device.

First, the 500 nm gold was sputtered onto a 500 μm thickness commercially purchased sapphire substrate by an electro-beam evaporation process, followed by an etching technology to form the designed resonant cells and electrodes. Then a 500 nm thickness vanadium dioxide (VO_2) film was grown on the sample by a radio frequency plasma-assisted oxide molecular beam epitaxy chamber. The VO_2 bridges were fabricated by tailoring the VO_2 layer with UV lithography technology, and the excess VO_2 was removed through a reactive ion etching procedure.[1] Next, a 300 nm Ge film was thermally sputtered onto the device surface. Finally, the redundant Ge was removed using a second UV lithography and lift-off technology.[2] The proposed terahertz metasurface can be prepared by the above process.

1. Lou, J.; Xu, X.; Huang, Y.D.; Yu, Y.; Wang, J.; Fang, G.Y.; Liang, J.G.; Fan, C.H.; Chang, C. Optically Controlled Ultrafast Terahertz Metadevices with Ultralow Pump Threshold. Small 2021, 17, 2104275.
2. Hu, Y.Z.; Tong, M.Y.; Xu, Z.J.; Cheng, X.G.; Jiang, T. Spatiotemporal Terahertz Metasurfaces for Ultrafast All-Optical Switching with Electric-Triggered Bistability. Laser Photonics Rev. 2021, 15, 2000456.
